# Engineering the Universal Donor: CRISPR-Mediated Blood Group Antigen Deletion and the Path Toward Truly Universal Red Blood Cells—A Narrative Review

**DOI:** 10.3390/diagnostics16152448

**Published:** 2026-08-03

**Authors:** Malik A. Altayar

**Affiliations:** Department of Medical Laboratory Technology, Faculty of Applied Medical Sciences, University of Tabuk, Tabuk 71491, Saudi Arabia; maltayar@ut.edu.sa

**Keywords:** universal donor blood, CRISPR-Cas9, blood group antigens, red blood cell engineering, alloimmunization, iPSC-derived red cells, enzymatic conversion, transfusion medicine

## Abstract

The designation of O RhD-negative blood as a “universal donor” misrepresents the complexity of red blood cell (RBC) compatibility. With 47 recognized blood group systems encompassing 366 antigens, non-ABO antigen exposure drives alloimmunization in 20–50% of chronically transfused patients. A narrative review of PubMed, Scopus, and EMBASE was conducted for studies published between 2016 and 2025, with selective inclusion of foundational pre-2016 works. Enzymatic approaches using Flavonifractor plautii CAZymes and Akkermansia muciniphila exoglycosidase cocktails enable near-complete ABO antigen removal, though group A conversion remains incomplete. CRISPR-Cas9 multiplex editing of immortalized erythroblasts has achieved simultaneous deletion of ABO, Rh, Kell, Duffy, and MNS antigens. Induced pluripotent stem cell platforms enable scalable manufacture; however, enucleation efficiency (40–70%), yield (4.6 × 10^3^ RBCs/iPSC), and fetal hemoglobin prevalence remain translational barriers. Convergence of enzymatic and gene-editing technologies on GMP-compatible iPSC platforms represents the most credible pathway to truly universal RBCs, although current evidence remains largely confined to immortalized cell lines and early-stage iPSC proof-of-concept studies rather than clinically validated products. Regulatory frameworks, scalability, and rigorous off-target profiling define the key challenges ahead.

## 1. Introduction

### 1.1. Importance of Universal Blood in Transfusion Medicine

Red blood cell (RBC) transfusion is among the most frequently performed medical procedures worldwide, providing lifesaving support for trauma, surgical blood loss, hematological malignancy, and chronic hemoglobinopathies. The World Health Organization recommends a minimum of 10–20 donations per 1000 population to meet basic healthcare needs; yet high-income countries achieve 31.5 donations per 1000, while low-income countries record only 5.0 per 1000—a disparity that translates into millions of preventable deaths annually [1].

Beyond absolute shortage, the logistical challenge of blood typing, cross-matching, and compatibility testing imposes critical time delays in emergency settings. A universal donor blood product—one compatible with all recipients regardless of blood group—would eliminate this bottleneck and transform transfusion medicine globally, particularly in resource-limited settings [2,3].

### 1.2. Limitations of Current ABO-Based Blood Supply

Blood inventory management is organized primarily around the ABO and Rh(D) systems, and the O RhD-negative phenotype is conventionally designated the “universal donor.” This heuristic is deeply embedded in clinical practice; however, it represents a significant scientific oversimplification. The International Society of Blood Transfusion (ISBT) recognizes 47 distinct blood group systems containing 366 RBC antigens as of October 2024, all genetically determined and immunologically relevant [4].

O-negative individuals represent only 6–8% of most donor populations, yet O-negative units are consumed disproportionately in emergencies, leading to perpetual scarcity. The COVID-19 pandemic illustrated the fragility of blood supply chains, with multiple countries experiencing acute shortages due to donor cancellations [5,6].

### 1.3. Clinical Need During Emergencies and Mass Casualty Events

In mass casualty events (MCEs) and pre-hospital settings, rapid blood transfusion often precedes formal ABO typing. Recent modeling studies suggest approximately 2000 units of blood per 100,000 people are required to meet routine demand; in MCEs this spikes sharply and unpredictably. Low- and middle-income countries, which bear the highest burden of traumatic injury, have the least reliable access to cross-matched blood.

### 1.4. Concept of Universal Donor Blood

**Figure 1:**
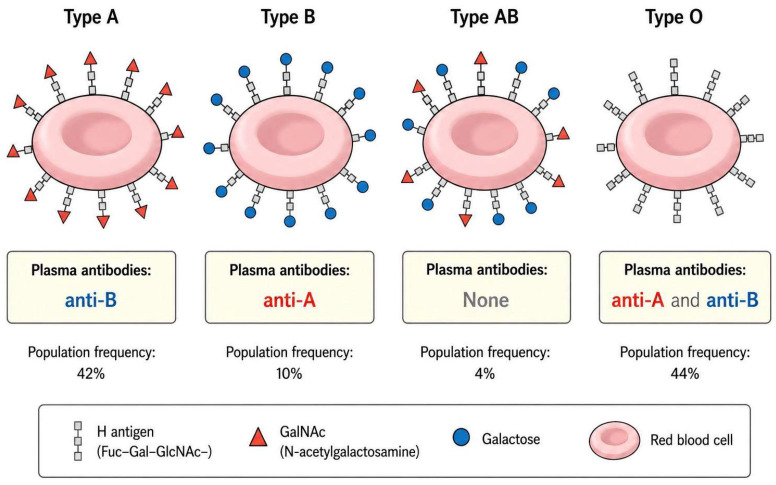
ABO Blood Group System—Antigen Structures, Plasma Antibodies, and Population Frequencies. Type A red blood cells express N-acetylgalactosamine (GalNAc) on the H antigen backbone; Type B express galactose; Type AB express both; Type O express neither. Corresponding plasma antibodies are shown below each cell type. GalNAc = N-acetylgalactosamine.

**Figure 2:**
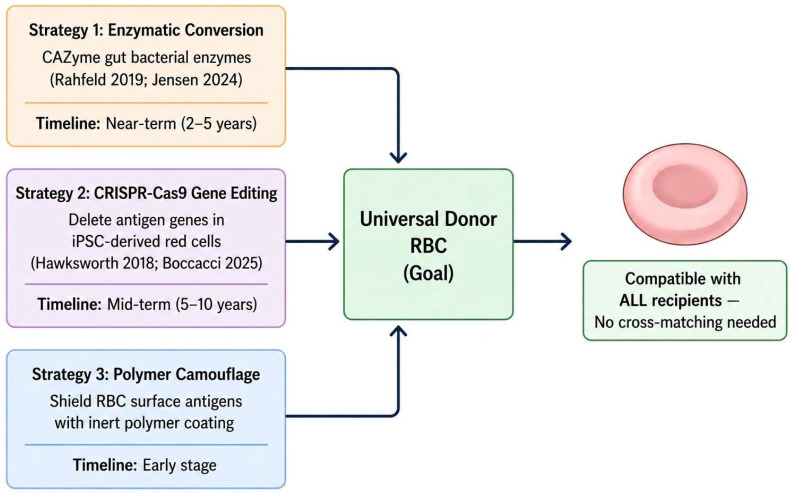
Three Principal Engineering Strategies Toward Truly Universal Red Blood Cells. Strategy 1 (enzymatic conversion) targets ABO antigens using carbohydrate-active enzyme (CAZyme) cocktails from gut microbiota; Strategy 2 (CRISPR-Cas9 gene editing) permanently deletes antigen-encoding genes in iPSC-derived red cells; Strategy 3 (polymer camouflage) shields surface antigens with inert polymer coating. Timelines reflect current estimated translational readiness.

The ideal universal RBC would carry no surface antigens capable of eliciting an alloimmune response in any recipient. For clarity, this review distinguishes two levels of “universality” throughout: ABO-universal denotes compatibility limited to the ABO system (i.e., a functional group O phenotype), whereas pan-compatible (used here interchangeably with “truly universal”) denotes the additional removal or masking of clinically significant non-ABO antigens (Rh, Kell, Kidd, Duffy, MNS, and others). These terms are not interchangeable and are used precisely throughout this review. Three broad strategies have been pursued: (1) enzymatic removal of blood group antigens from donor RBCs; (2) CRISPR-Cas9-mediated deletion of antigen-encoding genes in erythroblast lines or induced pluripotent stem cells (iPSCs); and (3) polymer camouflage to shield surface antigens. Of these, enzymatic and gene-editing approaches have shown the most substantive scientific progress [7,8,9,10,11,12]. The ABO antigen structures, plasma antibodies, and population frequencies that underlie this compatibility problem are shown in Figure 1, and the three engineering strategies are summarized schematically in Figure 2.

### 1.5. Literature Search Strategy

Literature was identified through PubMed, Scopus, and EMBASE for the period January 2016–2025, with selective inclusion of foundational studies published before 2016. Search terms combined the following concept groups using Boolean operators: (1) “universal donor blood” OR “universal red blood cells” OR “pan-compatible red blood cells”; (2) “enzymatic ABO conversion” OR “exoglycosidase” OR “blood group antigen removal”; (3) (“CRISPR-Cas9” OR “gene editing”) AND (“erythroid” OR “blood group antigen” OR “red blood cell”); (4) (“induced pluripotent stem cell” OR “iPSC”) AND (“erythropoiesis” OR “red blood cell”); (5) “alloimmunization” AND “transfusion”; and (6) “Rh blood group” OR “Kell blood group” OR “Duffy blood group” OR “Kidd blood group” OR “MNS blood group”. Reference lists of key articles were also hand-searched for additional relevant studies.

## 2. Beyond ABO: What Does “Universal” Really Mean?

### 2.1. Historical Concept of O-Negative Blood

The discovery of the ABO blood group system by Karl Landsteiner in 1901 established the scientific foundation for safe transfusion medicine. For much of the twentieth century, ABO and Rh(D) compatibility was considered sufficient. The designation of O RhD-negative blood as a “universal donor” emerged from this framework: type O individuals lack A and B antigens and cannot elicit ABO-mediated hemolytic reactions [13,14].

This heuristic served adequately when repeated transfusions were uncommon and patient populations were less immunologically diverse. However, the twentieth century brought an explosion of chronic transfusion requirements—sickle cell disease, thalassemia, myelodysplastic syndrome, cancer chemotherapy—for which the O-negative simplification proved increasingly inadequate [15,16].

### 2.2. Why O-Negative Is Not Truly Universal

Beyond ABO and Rh(D), a recipient can mount a clinically significant alloimmune response to any foreign antigen encountered. In sickle cell disease cohorts—who receive the majority of chronic transfusions—alloimmunization rates of 18–76% have been reported despite prophylactic Rh and Kell matching [17,18,19].

Each alloantibody formed complicates subsequent transfusion matching, increases the risk of delayed hemolytic transfusion reactions, and may render the patient essentially untransfusable when antibodies develop to high-frequency antigens. For this reason, a truly universal RBC must address not only ABO and Rh(D) but all clinically significant non-ABO antigen systems [20].

### 2.3. Role of Non-ABO Blood Group Systems

As of October 2024, ISBT recognizes 47 blood group systems determined by 52 genes, encoding 366 distinct antigens. The most clinically significant systems beyond ABO are Rh, Kell, Kidd, Duffy, and MNS, each presenting a distinct immunological and physiological profile that must be considered in designing a universal RBC. The Rh system alone comprises over 50 antigens and remains the most clinically dangerous after ABO [21].

#### 2.3.1. Rh System

The Rh system comprises more than 50 antigens encoded by two closely linked genes, RHD and RHCE, on chromosome 1p36. The five major antigens—D, C, c, E, and e—account for the majority of clinically significant alloimmunization [22,23]. Rh antibodies account for the majority of alloantibodies in sickle cell disease patients, as the higher prevalence of C, E, and K antigens in donor populations (predominantly of European ancestry) compared to recipient populations (predominantly of African ancestry) creates a systematic antigen mismatch.

**Figure 3:**
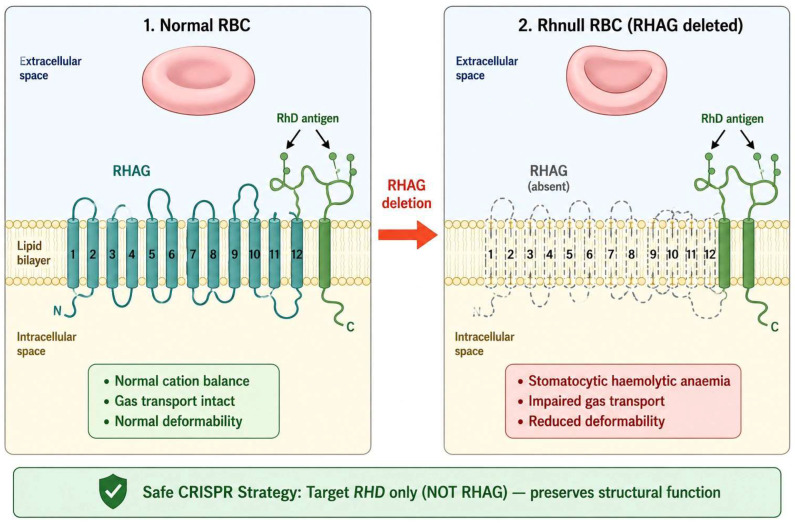
Rhnull Phenotype—Consequences of RHAG Deletion and Safe CRISPR Editing Strategy. Panel **1** (**left**): Normal RBC showing RHAG (12-pass transmembrane protein, teal) and RHD protein with RhD antigen sites; normal biconcave disk morphology. Panel **2** (**right**): Rhnull RBC following RHAG deletion—stomatocytic morphology, impaired cation balance and gas transport, reduced deformability. The red arrow indicates RHAG deletion. Bottom banner: Safe CRISPR strategy targets RHD exon 2 only, preserving RHAG structural function. N = amino terminus; C = carboxyl terminus.

The Rhnull phenotype—absence of all Rh antigens—is extraordinarily rare (estimated 1 in 600,000). Individuals with this phenotype due to RHAG deletion suffer from chronic stomatocytic hemolytic anemia, demonstrating that Rh proteins serve critical structural and physiological functions beyond antigenicity—a constraint that directly shapes the editing strategy for this system. This contrast between the normal RBC and the Rhnull phenotype, together with the safe RHD-restricted CRISPR editing strategy, is illustrated in Figure 3.

#### 2.3.2. Kell System

The Kell blood group system is encoded by the KEL gene on chromosome 7q34. The K antigen is expressed in only 9% of the Caucasian population and is highly immunogenic—a single K-positive exposure in a K-negative individual carries an immunization rate of approximately 10%, comparable to Rh(D) [24,25]. Anti-K is the most common non-ABO, non-Rh alloantibody and is associated with severe hemolytic disease of the fetus and newborn.

**Figure 4:**
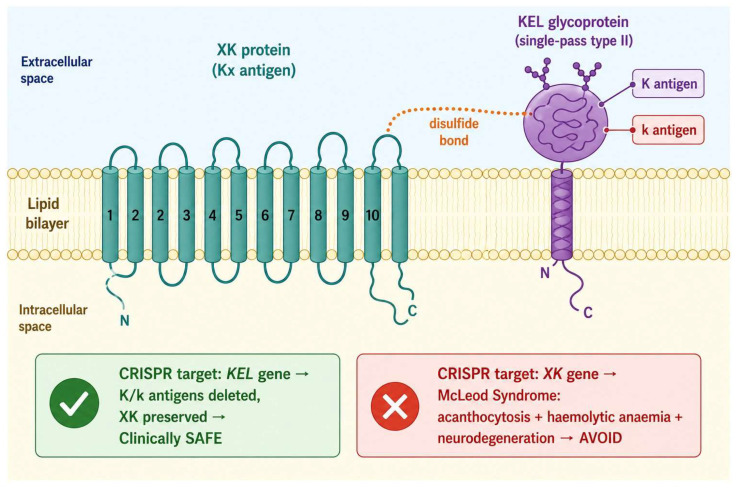
Kell Blood Group System—KEL/XK Protein Complex and McLeod Syndrome Constraint. XK protein (teal, 10-pass transmembrane) and KEL glycoprotein (purple, single-pass type II) are linked by a disulfide bond (orange dotted line). K and k antigens are located on the extracellular KEL domain. Lower panels show CRISPR targeting consequences: KEL gene targeting is clinically safe (green); XK gene targeting causes McLeod Syndrome and must be avoided (red). N = amino terminus; C = carboxyl terminus.

XK protein forms a complex with the Kell glycoprotein; disruption of XK results in McLeod syndrome—characterized by acanthocytosis, hemolytic anemia, and progressive neuromuscular degeneration. This prohibits CRISPR targeting of XK, constraining multiplex editing strategies to the KEL gene itself—a physiological constraint well-documented in the blood group genetics literature. This KEL/XK protein complex and the McLeod syndrome constraint are illustrated in Figure 4.

#### 2.3.3. Kidd System

**Figure 5:**
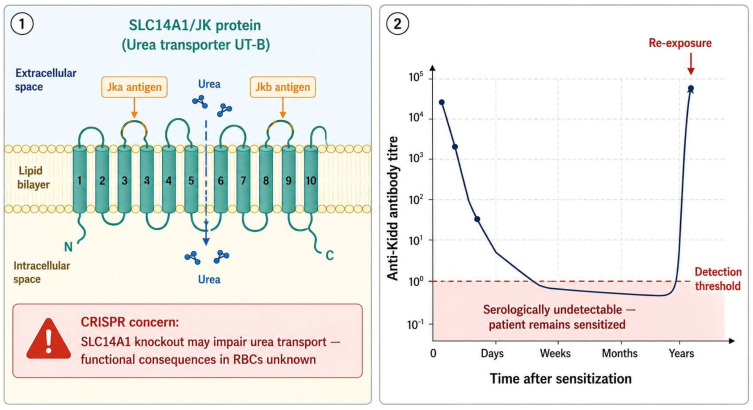
Kidd Blood Group System—SLC14A1 Dual Function and Anti-Kidd Antibody Waning Behavior. Panel **1** (**left**): SLC14A1/JK protein (teal, 10-pass transmembrane) showing Jka and Jkb antigen sites on extracellular loops and central urea transport channel (UT-B). Warning box: SLC14A1 knockout may impair urea transport—functional consequences in RBCs unknown. Panel **2** (**right**): Semi-logarithmic plot of anti-Kidd antibody titre over time showing rapid waning below the detection threshold (red dashed line) followed by anamnestic spike upon re-exposure. The shaded zone represents the clinically dangerous serologically undetectable period. N = amino terminus; C = carboxyl terminus.

The Kidd blood group system is encoded by the SLC14A1 gene, which also encodes the urea transporter UT-B. Kidd antibodies are particularly treacherous clinically because they wane rapidly after sensitization, falling below detection thresholds, only to resurge with subsequent antigen exposure and cause life-threatening delayed hemolytic transfusion reactions [20,24]. This dual function of SLC14A1 and the characteristic waning behavior of anti-Kidd antibodies are illustrated in Figure 5.

#### 2.3.4. Duffy System

**Figure 6:**
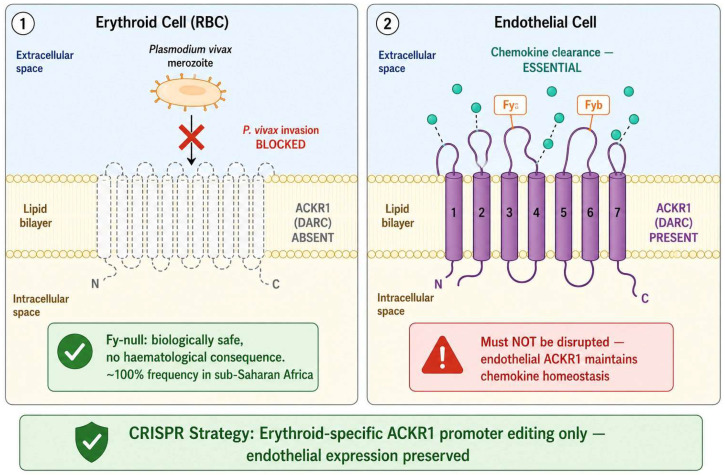
Duffy/ACKR1 Blood Group System—Differential Expression in Erythroid and Endothelial Cells. Panel **1** (**left**): Erythroid cell (RBC) showing ACKR1 absent (Fy-null phenotype)—Plasmodium vivax invasion is blocked; this phenotype is biologically safe and occurs at ~100% frequency in sub-Saharan Africa. Panel **2** (**right**): Endothelial cell showing ACKR1/DARC present (7-pass transmembrane, purple) with Fya and Fyb antigen sites and active chemokine clearance function. Bottom banner: Safe CRISPR strategy uses erythroid-specific ACKR1 promoter editing, preserving endothelial expression. N = amino terminus; C = carboxyl terminus.

The Duffy blood group system is encoded by ACKR1 (Duffy Antigen Receptor for Chemokines, DARC). The Fy(null) phenotype—complete absence of Duffy antigen on erythrocytes—is fixed at nearly 100% frequency in sub-Saharan African populations due to a promoter mutation (FY*O allele) that confers near-complete resistance to Plasmodium vivax malaria invasion [26,27]. Deletion of ACKR1 in a universal RBC product would produce a Duffy-null phenotype biologically well-tolerated in erythrocytes, but the DARC protein on vascular endothelium retains chemokine-clearing functions that must not be disrupted by erythroid-lineage-specific editing [28]. This differential ACKR1 expression and function in erythroid versus endothelial cells is illustrated in Figure 6.

#### 2.3.5. MNS System

The MNS blood group system is encoded by GYPA and GYPB, expressed as glycophorin A and glycophorin B. The S-s-U- phenotype, resulting from GYPB deletion, is found almost exclusively in individuals of African ancestry (approximately 1–2% prevalence) and is associated with anti-U antibody formation—a serious transfusion hazard for this population [29].

### 2.4. Alloimmunization Despite ABO Compatibility

A cross-system pattern emerges from examining these five antigen systems collectively: the clinical safety of engineering a null phenotype is not uniform but is determined by whether the target protein serves a structural or physiological function beyond antigen display. The Duffy-null phenotype is biologically safe in erythrocytes because ACKR1 is dispensable for normal RBC function, as evidenced by the large sub-Saharan African population carrying this phenotype without hematological consequence. By contrast, RHAG deletion causes stomatocytic hemolytic anemia because RHAG serves as a membrane channel essential for cation balance and gas transport, and XK deletion causes McLeod syndrome because XK anchors the Kell glycoprotein complex required for membrane integrity. This distinction—between antigen-display proteins whose absence is phenotypically silent and structural proteins whose loss is pathological—is the central biological constraint governing which null phenotypes can be safely engineered and which cannot. It also establishes a clear hierarchy of editing targets: ACKR1 promoter editing and GYPB deletion carry low physiological risk, and RHD-specific knockout is acceptable, but RHAG and XK must be preserved in any clinically viable multiplex editing strategy.

The aggregate burden of non-ABO alloimmunization is substantial. In the general transfusion-dependent population, alloimmunization rates range from 2 to 5%; in sickle cell disease this rises to 18–76% despite prophylactic Rh and K matching, reflecting substantial underlying genetic diversity in the variant alleles encoding these non-ABO antigens [30]. Each additional alloantibody reduces the pool of compatible donors exponentially [31,32].

**Figure 7:**
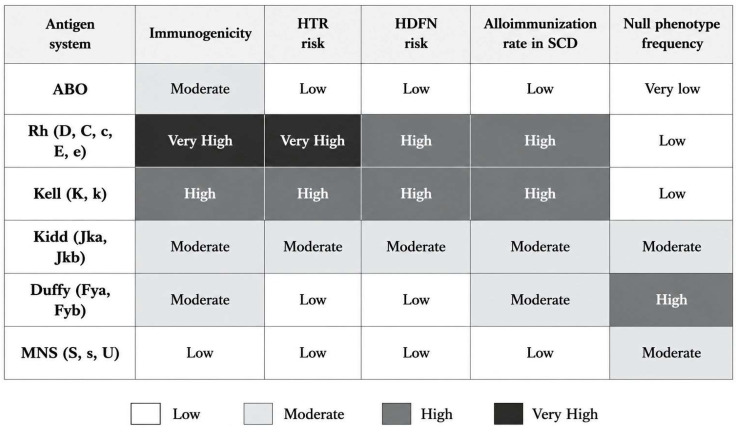
Clinical Significance Heatmap of Major Blood Group Antigen Systems. Shading intensity reflects risk level: white = Low; light gray = Moderate; dark gray = High; black = Very High. HTR = hemolytic transfusion reaction; HDFN = hemolytic disease of the fetus and newborn; SCD = sickle cell disease.

A critical synthesis emerges from this epidemiological data: the clinical demand for truly universal RBCs is driven primarily by the growing population of complex, chronically transfused patients for whom standard compatibility algorithms are failing. This population—comprising approximately 30% of all sickle cell disease patients, thalassemia patients, and patients with rare antibody combinations—represents the primary clinical target for engineered universal RBCs [33,34]. The clinical significance, alloimmunization risk, and CRISPR target genes for each of these blood group systems are summarized in Table 1, and the relative risk levels are presented visually as a heatmap in Figure 7.

## 3. Enzymatic Conversion Approaches

### 3.1. Concept of Enzymatic Antigen Removal

The principle of enzymatic blood group conversion is conceptually elegant: if the biochemical structures defining blood group antigens can be cleaved from RBC surfaces by specific exoglycosidases, the resulting cells would carry an O-type glycan profile and be immunologically transparent to all ABO-typed recipients. This idea was first demonstrated by Goldstein et al. in 1982, who used coffee bean alpha-galactosidase to partially convert B-type RBCs to a type-O-like phenotype [35,36]. While technically groundbreaking, this approach produced only partial conversion and targeted only the canonical B antigen.

Progress stalled for decades due to the difficulty of identifying enzymes with sufficient specificity and activity. The critical conceptual advance came from recognizing that ABO antigens on RBCs share structural similarity with mucin glycans in the human gut—redirecting the search toward the gut microbiome as a source of highly evolved, substrate-specific glycosidases [37,38].

### 3.2. CAZyme-Based ABO Conversion

The landmark study by Rahfeld and colleagues identified, through functional metagenomic screening of 19,500 expressed fosmids from gut bacterial DNA, an enzyme pair from Flavonifractor plautii that works in concert to convert A-type antigens to the H antigen. FpGalNAcDeAc first removes the acetyl group from the terminal N-acetylgalactosamine; FpGalNase then cleaves this intermediate to regenerate the H antigen. At very low enzyme concentrations, near-complete A-to-O conversion was achieved in whole blood.

The study by Jensen and colleagues, utilizing exoglycosidases from Akkermansia muciniphila, extended this principle to a far broader antigen target profile. By evaluating 23 Akkermansia glycosyl hydrolases, the researchers identified enzyme combinations capable of efficiently transforming not only canonical A and B antigens but also four of their carbohydrate extensions—including A type 3, H type 3, Gal-A, and ExtB. These extended variants had previously been a cause of unexplained incompatibility between enzymatically converted blood and type-O plasma. Enzymatic removal of both canonical and extended antigens significantly improved compatibility with group O plasmas.

**Figure 8:**
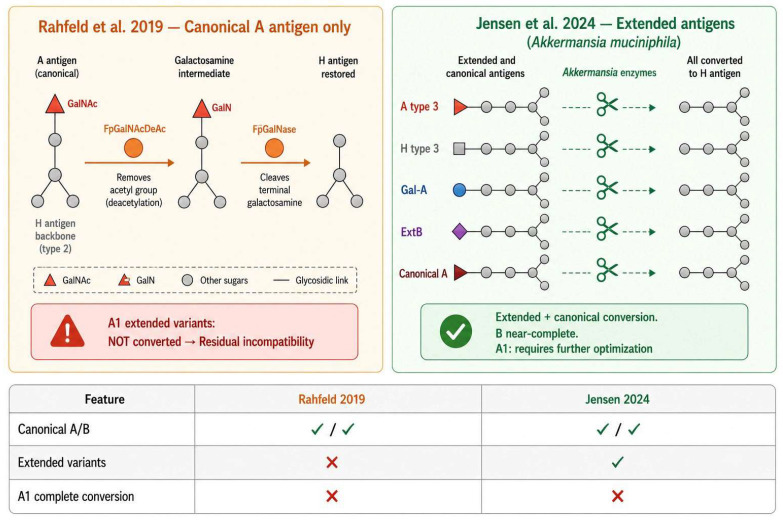
Comparison of Enzymatic ABO Conversion Approaches—Rahfeld et al. (2019) [10] versus Jensen et al. (2024) [11]. Left panel (orange): The Flavonifractor plautii two-enzyme CAZyme pair (FpGalNAcDeAc + FpGalNase) converts canonical A antigen to H antigen via a galactosamine intermediate; A1 extended variants are not converted. Right panel (green): Akkermansia muciniphila exoglycosidases convert five antigen variants (A type 3, H type 3, Gal-A, ExtB, Canonical A) to H antigen using scissor-action glycosidases. Comparison table (bottom) summarizes conversion scope. GalNAc = N-acetylgalactosamine; GalN = galactosamine.

The asymmetry between group B and group A conversion efficiency reveals an important mechanistic principle: the A antigen in the A1 subtype carries not only canonical N-acetylgalactosamine residues but also multiple extended carbohydrate branches that vary in accessibility and density, making complete cleavage substantially more difficult than for the B antigen. This is not merely a technical limitation of current enzyme cocktails—it reflects a fundamental biochemical asymmetry between the A and B antigens that has persisted across all generations of enzymatic conversion research since Goldstein and colleagues first described the approach in 1982. Resolving group A conversion will likely require structure-guided enzyme engineering that accounts for the three-dimensional presentation of extended A variants on the RBC surface, rather than incremental improvements to existing hydrolase activity. Taken together, the three limitations of enzymatic conversion—incomplete A1 conversion, ABO-only scope, and residual antigen detection—define the precise boundaries of what this approach can and cannot achieve: it is a powerful near-term tool for emergency ABO-universal blood production, not a solution to the broader challenge of pan-compatible transfusion. These major enzymatic ABO conversion approaches, their efficiency, and their key limitations are compared in Table 2, and the two leading approaches are compared mechanistically in Figure 8.

### 3.3. Limitations and Incomplete Conversion Concerns

Despite these advances, enzymatic conversion faces several important limitations. First, the current Akkermansia muciniphila enzyme cocktails achieve near-complete conversion for group B donors but require further optimization for group A blood, particularly the A1 subtype, which expresses higher antigen densities and a more complex array of extended variants [11,40].

Second, enzymatic conversion exclusively addresses ABO antigens and does not alter non-ABO blood group antigens—Rh, Kell, Kidd, Duffy, or MNS—which are responsible for the majority of clinically significant alloimmunization in chronically transfused populations. Enzymatically converted blood achieves “ABO-universal” status but falls short of being truly pan-compatible [41,42].

Third, the residual antigen detection threshold is a regulatory concern that has not been resolved. Patients who have previously formed anti-A antibodies may react to residual A antigen below the hemagglutination detection threshold [39,43]. This fundamental divergence—enzymatic conversion for ABO-incompatibility in acute-care and emergency transfusion settings versus CRISPR editing for pan-compatible RBCs in chronically transfused patients—is essential for rational prioritization of research investment.

The enzymatic conversion principle has also recently been extended beyond RBCs to solid-organ transplantation: enzymatic removal of blood group B antigens from a donor kidney during machine perfusion, followed by transplantation into a type O recipient with high-titre anti-B antibody, prevented hyperacute antibody-mediated rejection, with the allograft surviving 63 h before antigen re-expression, underscoring the broader translational momentum of this enzymatic strategy beyond transfusion medicine [44].

## 4. CRISPR-Cas9 Engineering of Universal Red Blood Cells

### 4.1. Mechanism of CRISPR-Mediated Antigen Deletion

The CRISPR-Cas9 system enables programmable, site-specific double-strand DNA cleavage guided by a single guide RNA (sgRNA). Upon recognition of a protospacer adjacent motif (PAM), Cas9 induces a double-strand break, which is repaired by either non-homologous end joining (NHEJ)—introducing indels that disrupt gene function—or homology-directed repair (HDR), enabling precise sequence substitution [45,46,47].

**Figure 9:**
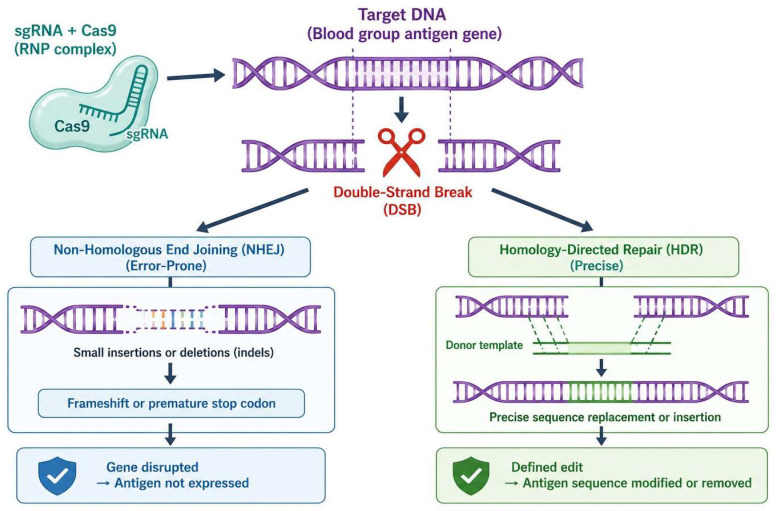
CRISPR-Cas9 Mechanism—Non-Homologous End Joining (NHEJ) versus Homology-Directed Repair (HDR). The sgRNA-Cas9 ribonucleoprotein (RNP) complex binds target DNA and induces a double-strand break (DSB, red scissors). NHEJ (left, blue): Error-prone repair introduces indels causing frameshift or premature stop codon—gene disrupted, antigen not expressed; used for ABO, KEL, ACKR1, and GYPB knockout. HDR (right, green): Precise repair using a donor template enables defined sequence replacement—antigen sequence modified or removed; used for RHD exon 2 editing to avoid RHCE off-target cleavage. RNP = ribonucleoprotein; DSB = double-strand break; indels = insertions/deletions.

In blood group engineering, NHEJ-based CRISPR editing introduces frameshift mutations that abolish gene expression, effectively deleting the antigen from the RBC surface. This approach was definitively demonstrated in 2018 by Hawksworth and colleagues—who simultaneously edited five blood group genes in the BEL-A erythroblast cell line to produce reticulocytes lacking ABO, Rh, Kell, Duffy, and MNS antigens—the first proof-of-concept of combinatorial multiplex blood group editing. The NHEJ and HDR repair mechanisms underlying these editing strategies are compared in Figure 9.

### 4.2. ABO Locus Editing

The ABO gene, located on chromosome 9q34, encodes a glycosyltransferase that catalyzes addition of sugar residues to the H antigen. Deletion or disruption of exons 6 or 7 by CRISPR-Cas9 abolishes transferase activity, producing cells that carry only the H antigen—the equivalent of blood group O. In 2022, Petazzi and colleagues demonstrated a particularly elegant application: using CRISPR/Cas9-mediated ABO gene editing to convert a type A iPSC line, derived from an Rhnull donor, to a universal type O phenotype—providing a paradigm for converting rare-donor iPSC lines to ABO-universal while preserving the rare phenotypic characteristics that make them valuable.

Boccacci and colleagues extended this approach, achieving near-complete ABO and Rh antigen knockout in HSPCs using a virus-free, selection-free CRISPR-Cas9 strategy, significantly reducing the genotoxic burden associated with viral vector delivery.

### 4.3. RhD and Extended Rh System Editing

RhD editing presents a particular challenge due to the 97% sequence homology between RHD and RHCE, which makes standard sgRNA design prone to inadvertent RHCE cleavage. Xu and colleagues addressed this by using HDR-based CRISPR/Cas9 with a donor template to introduce a premature stop codon specifically into RHD exon 2 of a hematopoietic-preferential hiPSC line [48]. The authors chose HDR over NHEJ precisely to avoid heterogeneous clone formation—a clinically critical consideration, as a seed cell population comprising both RhD-positive and RhD-negative cells would generate a mixed-antigen product unsuitable for transfusion.

A key insight from comparing Rh editing studies: RHAG-based targeting would produce a true Rhnull phenotype but carries unacceptable clinical risk because RHAG deletion impairs gas transport, cation balance, and membrane deformability. RHD-specific knockout removes the most clinically dangerous Rh antigen while preserving all structural functions, as the same study demonstrated.

### 4.4. Kell, Duffy, Kidd, and MNS System Editing

Beyond ABO and Rh, the Hawksworth multiplex strategy targeted three additional antigen systems. For the Kell system, CRISPR disruption of KEL (not XK) eliminates K and k antigen expression while preserving XK protein function and avoiding McLeod syndrome. For the Duffy system, ACKR1 editing replicates the naturally occurring Fy(null) phenotype found in populations of African ancestry—a phenotype with decades of observational evidence demonstrating erythrocyte safety. For the MNS system, GYPB deletion creates the S-s-U- phenotype, increasing compatibility with African-ancestry recipients who represent the most transfusion-dependent patient population [49].

The editing of the Kidd system (SLC14A1) is technically feasible but carries a physiological concern: SLC14A1 also encodes the urea transporter UT-B, and disruption may impair urea flux across the RBC membrane. No published study has yet reported the functional consequences of SLC14A1 knockout in iPSC-derived RBCs—a critical knowledge gap that must be addressed before clinical translation.

### 4.5. Multiplex Gene Editing—Toward Pan-Compatible RBCs

The proof-of-concept multiplex editing study demonstrated that simultaneous five-gene knockout in BEL-A cells produces viable, deformable reticulocytes with null phenotypes for all five targeted systems. This result demonstrates that multiple simultaneously edited loci do not result in cumulative cytotoxicity or disruption of erythroid differentiation—a non-trivial biological question resolved by Hawksworth and colleagues [7]. The five-antigen combination (ABO-null, RhD-null [RhD-negative; this study targeted RHD only and does not represent a true Rhnull phenotype, which requires RHAG disruption], K0, Duffynull, S-s-U-) is estimated to cover approximately 54 of 56 common blood donor genotypes, meaning these cells could theoretically be transfused to the vast majority of the global population without ABO or non-ABO alloimmunization risk [7].

However, a critical limitation inadequately highlighted in subsequent literature is the BEL-A cell platform: immortalized lines carry chromosomal abnormalities inherent to immortalization and are genomically unstable in ways that affect the fidelity of off-target analysis and the clinical safety profile [50]. The step from BEL-A proof-of-concept to iPSC-based clinical product requires independent validation of every null phenotype combination and comprehensive genome-wide off-target assessment in a clinically appropriate cell background.

Recent methodological work is beginning to address the editing-precision component of this limitation, even as the underlying genomic instability of immortalized lines persists: Daniels and colleagues systematically optimized ribonucleoprotein-based CRISPR-Cas9 delivery in BEL-A cells, achieving 48% precise gene editing efficiency for a defined point mutation, more than double the 22% biallelic efficiency previously reported for the same mutation in a comparable erythroid line (HUDEP-2) [51]. This improvement in editing precision, while valuable, does not resolve the chromosomal-abnormality concern intrinsic to the BEL-A platform itself.

### 4.6. iPSC-Derived RBCs as an Editing Platform

Induced pluripotent stem cells represent the most promising cellular platform for clinical manufacture of gene-edited universal RBCs, offering unlimited self-renewal, defined genetic background, and compatibility with GMP manufacturing [52,53]. The iPSC platform enables integration of CRISPR editing with manufacturing optimization—a convergence demonstrated by Varga and colleagues in their 2025 study describing a GMP-compatible dynamic culture system yielding 4.6 × 10^3^ RBCs per iPSC with 40–70% enucleation efficiency [54].

**Figure 10:**
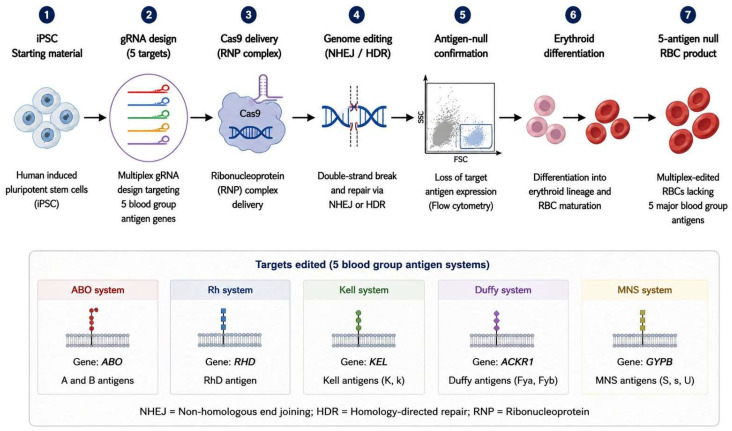
CRISPR-Cas9 Multiplex Blood Group Antigen Editing Workflow. Seven-step process from iPSC starting material to 5-antigen null RBC product. Multiplex guide RNAs targeting five blood group antigen genes (ABO, RHD, KEL, ACKR1, GYPB) are delivered as ribonucleoprotein (RNP) complexes to minimize off-target activity. NHEJ = non-homologous end joining; HDR = homology-directed repair; FSC = forward scatter; SSC = side scatter.

However, iPSC-derived RBCs consistently produce primitive-type erythropoiesis, characterized by high fetal hemoglobin (HbF, 60–80%) and lower oxygen-carrying capacity compared to adult RBCs. Strategies to promote adult-type maturation include use of immortalized lines derived from BM CD34+ cells, which more closely mimic adult erythropoiesis; BCL11A silencing, by contrast, increases rather than decreases HbF expression and is therefore not an appropriate strategy for this purpose [55,56]. The key CRISPR gene editing studies discussed throughout this section, including their target genes, platforms, and main findings, are summarized in Table 3, and the multiplex editing workflow from iPSC starting material to the 5-antigen null RBC product is illustrated in Figure 10.

A concise side-by-side comparison of the two leading engineering strategies described above—antigen coverage, evidence level, key limitations, manufacturing requirements, and likely clinical application—is provided in Table 4.

## 5. Safety, Immunological, and Regulatory Considerations

### 5.1. Off-Target Effects and Genotoxicity

Off-target activity (OTA) occurs when the Cas9-sgRNA complex binds to genomic loci with sequence similarity to the intended target, inducing double-strand breaks at unintended sites [57,58]. The consequences range from silent intronic insertions to disruption of tumor suppressor genes, with theoretically oncogenic potential.

Evidence has emerged of larger structural consequences of CRISPR-mediated DSBs not detectable by standard amplicon sequencing. Kosicki and colleagues demonstrated large deletions and complex rearrangements at on-target sites; Leibowitz and colleagues identified chromothripsis—massive chromosomal rearrangements—as an on-target consequence of Cas9 cleavage; and Nahmad and colleagues reported frequent aneuploidy in primary human T-cells following CRISPR-Cas9 cutting [59,60,61]. These findings demonstrate that the genotoxic footprint of CRISPR is substantially larger than originally appreciated.

Taken together, these three lines of evidence—large on-target deletions, chromothripsis, and aneuploidy—reveal a convergent and deeply troubling pattern: every major advancement in characterizing CRISPR genotoxicity has expanded, not contracted, the known risk profile. Each study identified a class of genomic damage that standard amplicon-sequencing-based off-target assays are structurally incapable of detecting. This progressive expansion of the known risk landscape suggests that current off-target profiling methods are not approaching a complete picture of CRISPR-mediated genome instability—they are capturing only the fraction of damage for which assays have been designed. For the field of universal RBC engineering, this has a direct implication: any pre-clinical safety package that relies solely on amplicon sequencing or predicted off-target site analysis will systematically underestimate genotoxic risk, and regulatory agencies are increasingly aware of this gap. Whole-genome sequencing, cytogenetic analysis, and long-read sequencing capable of detecting large structural variants must be considered the minimum acceptable standard for off-target profiling in any edited RBC product intended for human administration.

For universal RBC manufacturing, the acceptable risk threshold is far lower than for gene therapy for inherited disease—edited RBCs will be transfused into patients who are not otherwise irreversibly ill. High-fidelity Cas9 variants and ribonucleoprotein (RNP) delivery reduce off-target activity and eliminate the risk of Cas9 genomic integration but have not yet been validated across the five-target multiplex configuration required for pan-compatible RBCs [62].

### 5.2. Immunogenicity of Edited Cells

Theoretically, null phenotypes created by CRISPR editing could present novel epitopes—altered structural conformations at the cell surface—that might elicit immune responses in recipients; this remains an unstudied risk that warrants dedicated immunogenicity screening before clinical translation. An additional immunological concern is residual Cas9 protein: although RNP delivery eliminates genomic Cas9 integration, transiently expressed Cas9 may sensitize the immune system, and standard quality control must include ultrasensitive Cas9 protein detection assays in the final edited cell product [63].

### 5.3. Regulatory Pathway and GMP Compliance

Gene-edited RBCs do not fit neatly into existing regulatory frameworks. In the EU, they would likely be classified as Advanced Therapy Medicinal Products (ATMPs) under Regulation EC 1394/2007 [64]; in the US, a Biologics License Application (BLA) would be required with extensive pre-clinical and clinical safety data. The approval of CASGEVY (exagamglogene autotemcel) in late 2023 for sickle cell disease by both the FDA and EMA provides the first regulatory precedent for a CRISPR-based hematopoietic cell product and offers important lessons for the regulatory pathway of edited RBCs. However, this precedent should be interpreted cautiously: CASGEVY is an autologous product, in which a patient’s own cells are edited ex vivo and reinfused into the same individual, whereas a universal donor RBC product would be allogeneic, manufactured from a single donor-independent cell source and administered to genetically unrelated recipients at population scale. Neither CASGEVY nor the RESTORE trial therefore directly establishes a regulatory or manufacturing pathway for an allogeneic, multiplex-edited, iPSC-derived RBC product, and this gap remains a substantive translational barrier.

The RESTORE clinical trial—launched by NHS Blood and Transplant and the University of Bristol in 2021—represents the first-ever human transfusion of laboratory-manufactured RBCs grown from adult donor stem cells [65]. Although RESTORE cells are not gene-edited, the trial establishes GMP-manufacturing feasibility and provides the foundational clinical safety data framework for more complex, gene-edited products.

### 5.4. Ethical Dimensions

The primary ethical case for universal RBC development is strong: it would reduce transfusion mortality in LMICs, eliminate alloimmunization complications in chronically transfused patients, and improve equity of access to safe blood products globally. However, the development of engineered blood raises concerns about commodification of blood supply, dependency on commercial platforms, and equity in access. The populations most likely to benefit—individuals of African ancestry with sickle cell disease and thalassemia—are also those least likely to access expensive advanced biological products without active public investment and intentional health equity policies. The safety, immunological, and regulatory considerations discussed across this section are summarized in Table 5.

## 6. Manufacturing Challenges and Scalability

### 6.1. Ex Vivo Erythropoiesis—Current State

Platforms currently in use include: (1) immortalized erythroblast lines (BEL-A and derivatives), offering unlimited proliferative capacity but carrying genomic abnormalities inherent to immortalization; (2) CD34+ progenitor cells from peripheral blood or umbilical cord blood, which generate adult-type RBCs but are finite and donor-dependent; and (3) iPSC-derived systems, which offer unlimited self-renewal and genetic flexibility [66,67,68].

The first clinical milestone was achieved by the RESTORE trial—a Phase 1 study by NHS Blood and Transplant and the University of Bristol—reporting in November 2022 the first human transfusion of laboratory-manufactured RBCs grown from adult donor stem cells. The trial compares the in vivo survival of manufactured RBCs against conventionally donated RBCs from the same donor, establishing that GMP-grade manufacturing of erythroid cells from adult stem cells is achievable, as the RESTORE trial demonstrated.

A complementary manufacturing advance directly targets the cytokine-dependency and culture-duration barriers that limit iPSC-derived RBC scale-up: Olivier and colleagues engineered iPSC lines carrying constitutive stem cell factor receptor and JAK2 adaptor alleles, enabling the resulting self-renewing erythroblasts to proliferate for up to 70 population doublings in a chemically defined, cytokine-free medium while retaining high-rate enucleation capacity [69]. This approach offers a potential route to substantially reduced production costs, one of the central barriers identified in Section 6.3.

### 6.2. Enucleation Efficiency and Yield

The most significant technical barrier to clinical-scale RBC manufacturing from iPSCs remains enucleation—the process by which erythroid precursors expel their nuclei. Unlike primary erythropoiesis in bone marrow, in vitro protocols must replicate these signals in a controlled bioreactor environment [70]. Current state-of-the-art dynamic culture systems achieve 40–70% enucleation, yielding approximately 4.6 × 10^3^ RBCs per iPSC—sufficient for a mini-transfusion unit from 4.9 × 10^7^ iPSCs, as Varga and colleagues reported.

An underappreciated mechanistic connection links developmental immaturity and low enucleation efficiency: both are manifestations of failure to fully recapitulate definitive hematopoiesis in vitro. Strategies that force definitive hematopoiesis—including c-kit ligand signaling manipulation and bone marrow-derived stromal coculture—target enucleation and hemoglobin switching as linked outputs of the same developmental program; BCL11A silencing, by contrast, promotes a fetal-type globin program and would not support this transition [71].

### 6.3. Cost-Effectiveness and Clinical Translation

**Figure 11:**
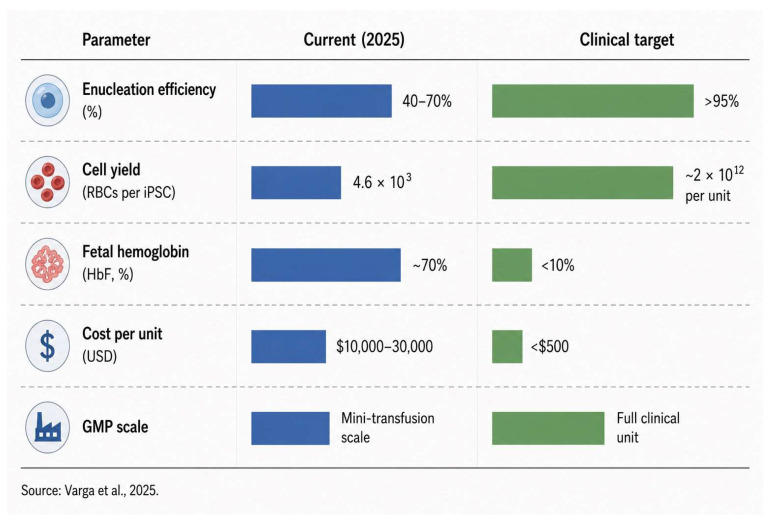
Current Status versus Clinical Target for Key iPSC-to-RBC Manufacturing Parameters (2025). Blue bars represent current achieved values; green bars represent minimum clinical translation targets. Data derived from Varga et al. (2025) [54]. HbF = fetal hemoglobin; GMP = good manufacturing practice; USD = United States Dollar.

Current cost estimates for iPSC-derived RBC production range from US$10,000 to US$30,000 per unit, compared to approximately US$200–300 per conventionally donated unit. A rational clinical translation strategy would not aim to replace the entire blood supply but rather target patients for whom the cost premium is medically justified: heavily alloimmunized patients with no compatible donor match, patients with multiple red cell alloantibodies requiring emergency transfusion, and patients with rare blood group phenotypes for whom no suitable donor exists. The key manufacturing challenges discussed throughout this section, including current status and clinical targets, are summarized in Table 6, and the gap between current achieved values and minimum clinical translation targets is shown graphically in Figure 11.

## 7. Discussion

This review has synthesized the current scientific evidence for enzymatic ABO conversion and CRISPR-Cas9 gene editing as pathways toward truly universal RBCs and has evaluated them within the clinical context of alloimmunization, global blood supply deficits, and iPSC-based manufacturing. Several overarching themes deserve explicit discussion.

First, the distinction between “ABO-universal” and “truly universal” has direct implications for which patient populations benefit and what level of scientific challenge must be surmounted. Enzymatic conversion achieves ABO-universality and represents a genuine near-term contribution to emergency transfusion logistics. CRISPR-mediated gene editing on iPSC platforms aims for pan-compatibility across all major antigen systems and targets the chronically transfused, heavily alloimmunized patient population [7,8,10,11,40]. These are complementary strategies with different timelines, risk profiles, and target populations.

Second, all published CRISPR proof-of-concept data are generated in immortalized cell lines (BEL-A) or proof-of-principle iPSC experiments—not in a manufacturing-grade, clinically compliant product. The BEL-A platform is genomically abnormal and cannot serve as a clinical product. The step from BEL-A proof-of-concept to GMP-grade iPSC clinical product requires re-establishing every multiplex phenotype combination with comprehensive off-target profiling in a validated clinical-grade cell background [59,60,61,63].

Third, the manufacturing barriers—enucleation efficiency, HbF prevalence, and yield—all trace to the same underlying problem of incomplete definitive hematopoiesis induction. Because BCL11A silencing increases rather than suppresses HbF expression, it is not a viable route to reducing fetal hemoglobin in this context; strategies that instead promote definitive, adult-type erythropoiesis—such as bone marrow-derived starting material and optimized stromal coculture—remain the more direct approaches to jointly addressing HbF prevalence, maturation, and enucleation as linked outputs of the same developmental program [52,54,56,71].

Fourth, the ethical and equity dimensions cannot be treated as peripheral concerns. The populations with the greatest unmet clinical need—chronically transfused patients with sickle cell disease and thalassemia, predominantly of African and South Asian ancestry—are precisely those most likely to be excluded from access to expensive advanced biological products without active public investment [1,2,3].

Finally, studies reporting “near-complete” antigen removal or “no detectable” off-target effects must be interpreted against the background of the detection threshold of their assays. Ultra-sensitive mass spectrometry-based antigen detection, whole-genome sequencing for structural variant analysis, and in vivo circulation studies in animal models represent the minimum evidential threshold for advancing any engineered universal RBC product to human trials [11,42,58,62].

## 8. Conclusions

The engineering of truly universal donor red blood cells represents one of the most scientifically ambitious and clinically impactful goals in contemporary transfusion medicine. This review has documented substantial progress on two fronts: enzymatic ABO conversion through Akkermansia muciniphila exoglycosidase cocktails, and CRISPR-Cas9 multiplex gene editing demonstrating simultaneous deletion of five major blood group antigen systems in erythroid cell lines. The convergence of CRISPR-based multiplex editing with iPSC-based manufacturing platforms—building on proof-of-concept gene-editing studies together with the manufacturing feasibility demonstrated by the non-gene-edited RESTORE clinical trial and advances in dynamic GMP-compatible bioreactor culture—provides a credible, though still preclinical, translational architecture.

However, honest assessment of the field reveals that the gap between proof-of-concept and clinical product is larger than is sometimes acknowledged. Off-target mutagenesis profiling remains inadequate, enucleation efficiency and cell yield remain below clinical thresholds, fetal hemoglobin predominance has not been resolved, and no regulatory pathway has been defined for a gene-edited RBC product. Addressing these challenges requires scientific innovation, collaborative regulatory science, sustained public investment, and explicit commitment to equity in access.

The vision of a universal donor blood product—one that can be stored, transported, and administered to any patient anywhere without compatibility testing—is achievable. But its realization will require the same scientific rigor, critical skepticism, and long-term commitment that have characterized the greatest achievements in transfusion medicine.

## Figures and Tables

**Table 1:** Blood Group Systems: Clinical Significance, Alloimmunization Risk, and CRISPR Target Genes.

System	Gene(s)	Key Antigens	Clinical Significance	Alloimmunization Risk	CRISPR Target
ABO	ABO	A, B, H	Immediate HTR; fatal if mismatched	Very High	ABO exon 6/7
Rh	RHD, RHCE	D, C, c, E, e	Delayed HTR; HDFN; second most immunogenic	High (anti-D: up to 80%)	RHD exon 2 (HDR)
Kell	KEL, XK	K, k, Kpa, Kpb	Severe HDFN; HTR; K highly immunogenic	High (~10% per K exposure)	KEL (avoid XK—McLeod risk)
Kidd	SLC14A1	Jka, Jkb	Antibodies wane; severe delayed HTR	Moderate	SLC14A1 (urea transport risk)
Duffy	ACKR1 (DARC)	Fya, Fyb	HTR; P. vivax invasion receptor	Moderate–High	ACKR1 promoter (erythroid-specific)
MNS	GYPA, GYPB	M, N, S, s, U	S–s–U– in Africans; anti-U is serious hazard	Moderate	GYPB deletion

Note. HTR = hemolytic transfusion reaction; HDFN = hemolytic disease of the fetus and newborn; HDR = homology-directed repair. Antigen frequencies and clinical risk data from Reid et al. 2012 [20] and Cohn et al. 2020 [25].

**Table 2:** Comparison of Major Enzymatic ABO Blood Group Conversion Approaches.

Approach	Source	Target Antigens	Efficiency	Key Limitation	Reference
Alpha-galactosidase (classical)	Coffee beans	B antigen (canonical only)	Partial	Extended B variants not removed	Goldstein et al. 1982
FpGalNAcDeAc + FpGalNase (CAZyme pair)	Flavonifractor plautii	A antigen (canonical)	Near-complete A-to-O at low enzyme conc.	Extended A variants (type 3) not targeted	Rahfeld et al. 2019
Akkermansia exoglycosidase cocktail	Akkermansia muciniphila	A, B + 4 carbohydrate extensions	B near-complete; A requires optimization	Group A1 conversion incomplete	Jensen et al. 2024
Structure-guided directed evolution	Engineered glycosidases	Extended A/B variants	Improved kcat/Km (in development)	Not yet clinical-grade	Kwan et al. 2015

Note. CAZyme = carbohydrate-active enzyme; conc. = concentration. References: Goldstein et al. 1982 [35], Rahfeld et al. 2019 [10], Jensen et al. 2024 [11], Kwan et al. 2015 [39].

**Table 3:** Summary of Key CRISPR Gene Editing Studies in Universal Red Blood Cell Engineering.

Study	Target Gene(s)	Platform	Strategy	Phenotype	Key Finding
Hawksworth et al. 2018 [7]	ABO, RHD, KEL, ACKR1, GYPB	BEL-A erythroblasts	Multiplex NHEJ KO	5-antigen null reticulocytes	First proof-of-concept multiplex editing; 54/56 donor genotypes covered
Petazzi et al. 2022 [9]	ABO (A-to-O)	Rhnull hiPSC	CRISPR/Cas9 HDR frameshift	Type O, Rhnull phenotype	Paradigm for converting rare-donor iPSCs to ABO-universal; applicable to non-O rare phenotypes
Xu et al. 2023 [48]	RHD exon 2	hiPSC (HuAiPSC)	HDR—premature stop codon	O-type RhD-negative erythrocytes	HDR prevents heterogeneous clones vs. NHEJ; optimized O2 differentiation improves yield
Boccacci et al. 2025 [8]	ABO + RHCE combined	Mobilized HSPCs	Virus-free, selection-free Cas9 KO	O-type and Rh-antigen-depleted (D/C/E-negative; not true Rhnull, as RHAG was not targeted)	Virus-free approach reduces genotoxic risk; combined ABO + Rh editing advances clinical feasibility
Varga et al. 2025 [54]	N/A (manufacturing)	iPSC-derived RBCs	Dynamic bioreactor GMP-compatible	40–70% enucleation; 4.6 × 10^3^ RBC/iPSC	GMP-compatible system; mini-transfusion unit feasibility (4.9 × 10^7^ iPSCs per unit)

Note. BEL-A = Bristol Erythroid Line Adult; NHEJ = non-homologous end joining; HDR = homology-directed repair; hiPSC = human induced pluripotent stem cell; HSPC = hematopoietic stem and progenitor cell; KO = knockout; GMP = good manufacturing practice. All studies PubMed/DOI-verified.

**Table 4:** Comparison of Enzymatic Conversion and CRISPR-Cas9 Gene Editing Approaches for Universal Red Blood Cell Engineering.

Approach	Antigen Coverage	Evidence Level	Key Limitations	Manufacturing Requirements	Likely Clinical Application
Enzymatic conversion (CAZyme/exoglycosidase cocktails)	ABO only (A/B antigens); does not address Rh, Kell, Kidd, Duffy, MNS, or other non-ABO systems	In vitro conversion of donor RBCs; near-complete for B antigen, incomplete for A antigen	Incomplete A-antigen conversion; residual antigen may retain immunogenicity; conversion must be repeated per donor unit rather than fixed at the donor level	Enzyme production/purification; conversion performed ex vivo on collected donor units; compatible with existing blood-bank infrastructure	Near-term emergency/trauma transfusion logistics; ABO-universal supply only
CRISPR-Cas9 multiplex gene editing (erythroblast lines/iPSC)	Potentially pan-compatible; proof-of-concept multiplex deletion of ABO, RHD, KEL, ACKR1, and GYPB demonstrated simultaneously	Proof-of-concept in immortalized cell lines (BEL-A) and early-stage iPSC systems; not yet validated in a GMP-grade clinical product	Off-target mutagenesis profiling incomplete; genomic instability of immortalized platforms; low enucleation efficiency and high HbF in iPSC-derived cells; no defined regulatory pathway	GMP-compatible iPSC expansion, multiplex editing, differentiation, and enucleation at clinical scale; substantially more complex and costly than enzymatic conversion	Longer-term durable solution for chronically transfused, heavily alloimmunized patients (e.g., sickle cell disease, thalassemia)

Note. CAZyme = carbohydrate-active enzyme; iPSC = induced pluripotent stem cell; BEL-A = Bristol Erythroid Line Adult; GMP = good manufacturing practice; HbF = fetal hemoglobin. Evidence levels reflect the developmental stage of the cited studies as of manuscript preparation and should not be read as claims of clinical validation.

**Table 5:** Safety, Immunological, and Regulatory Considerations for CRISPR-Edited Universal Red Blood Cells.

Safety Domain	Concern	Detection Method	Mitigation Strategy	Current Evidence
Off-target mutagenesis	Indels at unintended loci; oncogenic potential	GUIDE-seq, CIRCLE-seq, WGS	High-fidelity Cas9 variants (eSpCas9, HiFi Cas9)	No standardized guidelines; under regulatory scrutiny
Chromosomal instability	Aneuploidy, chromothripsis, large deletions	SNP arrays, WGS, cytogenetics	Base/prime editing (no DSB); reduce MOI	Reported in T-cells and HSCs; risk in erythroid progenitors under investigation
Immunogenicity of edited cells	Novel epitopes from null phenotypes; residual Cas9	Mixed lymphocyte reaction; murine in vivo models	RNP delivery to minimize Cas9 persistence	Limited direct clinical data for edited RBCs
Regulatory pathway	Classification as ATMPs/BLA; no RBC-specific framework	FDA/EMA ATMP classification review	Early regulator engagement; learn from CASGEVY 2023	CASGEVY approved 2023—precedent but not directly applicable to RBCs
Null phenotype physiology	McLeod syndrome (XK null); Rhnull stomatocytosis; DARC chemokine role	Ektacytometry; hematological assays	Selective KO: ACKR1 promoter only; avoid RHAG	XK KO avoided; RHAG deletion impairs gas transport and deformability

Note. ATMPs = Advanced Therapy Medicinal Products; BLA = Biologics License Application; DSB = double-strand break; MOI = multiplicity of infection; RNP = ribonucleoprotein; CASGEVY approved by FDA/EMA in 2023. References: Carusillo 2020 [62], Kosicki 2018 [59], Leibowitz 2021 [60], Nahmad 2022 [61], Levesque 2025 [63].

**Table 6:** Key Manufacturing Challenges for iPSC-Derived Universal Red Blood Cells: Current Status and Targets.

Challenge	Current Status	Clinical Target	Approach	Key Reference
Enucleation efficiency	40–70% (dynamic culture)	>95%	Macrophage coculture; OP9 stromal support; prolonged culture	Varga et al. 2025 [54]; Gunawardena et al. 2024 [70]
Cell yield	~4.6 × 10^3^ RBC/iPSC	~2 × 10^12^ per unit	Bioreactor scale-up; BEL-A immortalized lines	Varga et al. 2025 [54] ; Trakarnsanga et al. 2017 [55]
Fetal hemoglobin (HbF)	~60–80% HbF	<10% HbF	Definitive hematopoiesis induction (BM-derived starting material, stromal coculture)	Gunawardena et al. 2024 [70]
Cost per unit	~US$10,000–30,000	<US$500	Process automation; bioreactors; public subsidy models	Gunawardena et al. 2024 [70]; Focosi & Amabile 2018 [53]
GMP compliance	RESTORE trial: first human GMP trial (2022–ongoing)	Phase II/III approval	Feeder-free, xeno-free, validated single-supplier reagents	Toye et al. 2022 [65] ; Chukwuemeka 2025 [68]

Note. iPSC = induced pluripotent stem cell; RBC = red blood cell; HbF = fetal hemoglobin; GMP = good manufacturing practice; RESTORE = REcovery and Survival of STem cell Originated REd cells trial. Yield and enucleation data from Varga et al. 2025 [54]. Cost estimates from Gunawardena et al. 2024 [70] and Focosi & Amabile 2018 [53].

## Data Availability

No new data were created or analyzed in this study. Data sharing is not applicable to this article.
